# Association Between Serum Trace Elements Level and Alopecia Areata: A Systematic Review and Meta‐Analysis

**DOI:** 10.1111/jocd.16740

**Published:** 2024-12-30

**Authors:** Ruiying Wu, Yuanwen Li, Hongye Peng, Zhishan Yang, Ying Wang, Shuying Lv, Dingquan Yang

**Affiliations:** ^1^ School of Clinical Medicine Beijing University of Chinese Medicine Beijing China; ^2^ Department of Dermatology China‐Japan Friendship Hospital Beijing China; ^3^ National Medical Center for Integrated Chinese and Western Medicine Beijing China; ^4^ Department of Dermatology Beijing University of Chinese Medicine Dongfang Hospital Beijing China

**Keywords:** alopecia areata, meta‐analysis, serum trace element, vitamin D, zinc

## Abstract

**Background:**

Alopecia areata (AA) is a prevalent autoimmune disorder in dermatology, impacting 0.5%–2% of the general population worldwide. More and more scholars are focusing on the important role of micronutrients in the occurrence and development of AA.

**Aims:**

This research aimed to thoroughly and systematically assess the relationship between serum trace element levels and AA.

**Methods:**

The PubMed, Web of Science, EMBASE, and Cochrane Library databases were searched up to May 12, 2024. Two researchers independently screened and identified eligible studies. Depending on the heterogeneity assessed by the *I* ^2^ statistic, either a random‐effects model or a fixed‐effects model was used to combine the effect estimates.

**Results:**

34 papers, including 4931 participants from 16 countries, were analyzed. The results of meta‐analysis demonstrated patients with AA had a lower serum level of vitamin D (SMD = −0.93 ng/mL, 95% CI = 0.168–0.747, *p* < 0.05) and serum zinc (SMD = −0.69 μg/dL, 95% CI = −0.99 to −0.39, *p* < 0.05) than the healthy controls. Vitamin D deficiency was strongly associated to an elevated risk of AA (OR = 2.48, 95% CI = 1.47–4.17, *p* < 0.05). However, there is no significant difference in serum copper levels between AA patients and the control group.

**Conclusions:**

Our research provided evidence that the levels of serum VD and zinc were associated with the risk of AA. Supplementation with VD and zinc may become a potential treatment for AA.

## 1 Introduction

Alopecia areata (AA) is a prevalent autoimmune disorder in dermatology that is mainly manifested by the sudden appearance of round or oval patches of hair loss on the localized scalp or other hairy areas, and in severe cases, it can progress to total baldness and generalized baldness [1]. AA affects 0.5%–2% of the general population worldwide, and the incidence of the disease is becoming increasingly youthful [2]. AA not only affects the appearance of the patient, leading to psychological problems such as depression and anxiety, but also may increase the risk of thyroid disorders, autoimmune diseases such as systemic lupus erythematosus, and complications such as skin infections [3]. A survey estimated the total nationwide cost of AA at US$ 857 million in Japan, where the average monthly cost of medication for patients with AA was US$ 32.42 [4]. Therefore, the discovery of risk factors for AA and targeted interventions are essential for improving the quality of life of patients with AA and reducing the national economic burden.

However, the specific etiology of AA has not yet been fully clarified, and it is closely related to a variety of factors such as genetic factors, environmental factors, and mental stress [5]. More and more scholars are focusing on the important role of micronutrients in the occurrence and development of AA [6, 7, 8]. Zinc plays an important role in cell division and hair follicle growth, and it affects the growth and repair of the hair. Zinc deficiency may lead to impaired follicle function, which may lead to alopecia [9]. Lalosevic et al. [6] found that serum zinc concentrations were significantly lower in patients with AA compared to healthy controls. Copper is involved in angiogenesis and maintaining the integrity of the vessel wall, which helps to maintain a good blood supply to the hair follicle. In addition, copper is an essential cofactor for a number of enzymes (e.g., superoxide dismutase, tyrosinase, etc.), which play a key role in antioxidant protection, prevention of cellular damage, and melanogenesis. Studies have shown that serum copper levels in patients with alopecia totalis differ significantly from those in patients with AA [10]. Although Kil, Kim, and Kim [11] found that serum copper levels in patients with AA did not differ significantly from those in healthy controls. Vitamin D (VD), on the other hand, affects the growth cycle of the hair follicle through its binding to the VD receptor [12]. Thus, exploring the relationship between serum exposure to trace elements and the risk of AA is of great public health value. Although a few studies have been conducted to explore the relationship between the two, the overall number is limited. In this context, a synthesis and summary statistics of all the published literature could provide a higher level of evidence and scientific information.

To the best of our limited knowledge, only Jin et al. [13] conducted a meta‐analysis of the relationship between serum trace elements and the risk of AA, but that study did not explore VD. At the same time, the publication of numerous new clinical studies in recent years prompted us to conduct a systematic review and meta‐analysis of existing research. Our goal was to comprehensively and systematically assess the relationship between serum trace element levels (zinc, copper, and VD) and the risk of developing AA.

## 2 Methods

### 2.1 Search Strategy

The meta‐analysis adhered to the PRISMA guidelines [14] and included an extensive search of the PubMed, Web of Science, EMBASE, and Cochrane Library databases from their inception until May 12th 2024, using a combination of the following keywords: “trace element,” “zinc/Zn,” “copper,” “vitamin D/cholecalciferol/calciferol/25‐hydroxy vitamin D/25‐hydroxy cholecalciferol,” and “alopecia areata/ alopecia totalis/ alopecia universalis.” The detailed search strategy utilized for the four databases is available in Table S1. The meta‐analysis included only human studies, with no restrictions on language. Two researchers independently screened and included eligible studies, and any disagreements were resolved through discussion with a third researcher to ensure result consistency. The study protocol is registered on the PROSPERO website (http://www.crd.york.ac.uk/PROSPERO), CRD42024563741.

### 2.2 Inclusion Criteria and Exclusion Criteria

Studies fulfilling the following inclusion criteria were included: (1) The subjects were AA or healthy controls; (2) case–control studies or cross‐sectional or cohort; (3) studies were published in English; and (4) the observables were serum levels of trace element (Zn, Cu, and VD).

Duplicate papers that provide further information on previously listed research were eliminated. Additionally, the following types of studies were excluded: (1) animal studies; (2) reviews, case reports or not original studies; (3) subjects with diseases and drug intake that may affect serum trace element levels; and (4) inability to extract specific value.

### 2.3 Data Extraction and Quality Assessment

Important details from the publications were gathered separately by two researchers using a uniform form. The information collected included the first author's name, the year of publication, participant characteristics (gender, age, and nationality), sample size, type of serum trace element, study design, key findings, and any other relevant data. When an article was included but did not give standard mean difference (SMD), hazard ratio (HR), odds ratio (OR), or 95% confidence intervals (CIs) directly, we used the original data to generate these values. The SMD is used to compare the differences in average values (means) between two groups, standardized to account for variations in measurement scales. HR refers to the ratio of the risk of a specific endpoint event (such as disease progression, recurrence, or death) occurring within a certain period of time in a population exposed to a particular factor (such as a medication, treatment, or risk factor) compared to a population not exposed to that factor. It reflects the relative risk over time. OR represents the ratio of the “exposed to unexposed individuals” in the case group to the “exposed to unexposed individuals” in the control group. An OR value > 1 indicates that the factor is a risk factor, meaning that individuals exposed to this factor have an increased risk of developing the disease.

Cohort and case–control study quality was assessed using the Newcastle–Ottawa Scale (NOS). The total score is nine points, with seven points and above being high quality [15]. We used the Joanna Briggs Institute's (JBI) recommended scale to evaluate cross‐sectional studies (http://www.joannabriggs.edu.au/). The total score is 20 points, with 16 points and above being high quality [16].

### 2.4 Assessment of Heterogeneity

The appropriateness of merging the information from the included studies, such as publication year, sample size, male‐to‐female ratio, and study quality, was assessed clinically and methodologically. To evaluate statistical heterogeneity, forest plots were visually inspected and statistical techniques were used. The Cochran's *Q*‐test and the *I* ^2^ statistic were used to examine homogeneity [17]. High heterogeneity was defined as a value > 50% [18].

### 2.5 Statistical Analysis

*I* ^2^ ≥ 50% was deemed to be “high” heterogeneity for the purposes of the analysis, and the random effects model was used to combine the effect estimates. However, the fixed effects model was applied [19]. Measurements of the variations in blood trace element levels between the patients with AA and health controls were made using the SMD with 95% CI across studies. For serum VD, subgroup analyses were used based on publication year (before 2019 or after 2019), sample size (> 80 or ≤ 80), gender ratio (Male‐to‐female ratio > 1.5 or Male‐to‐female ratio < 1.5) and study quality (low or high). For serum Zinc, subgroup analyses were used based on publication year (before 2016 or after 2016), sample size (> 80 or ≤ 80), and study quality (low or high). Begg's test and Egger's test were used to evaluate potential publication bias at the *p* < 0.05 level of significance. Every statistical analysis was performed using Stata version 15 (Stata Corp., College Station, Texas).

## 3 Results

### 3.1 The Features of Literature Retrieval and Study

**FIGURE 1:**
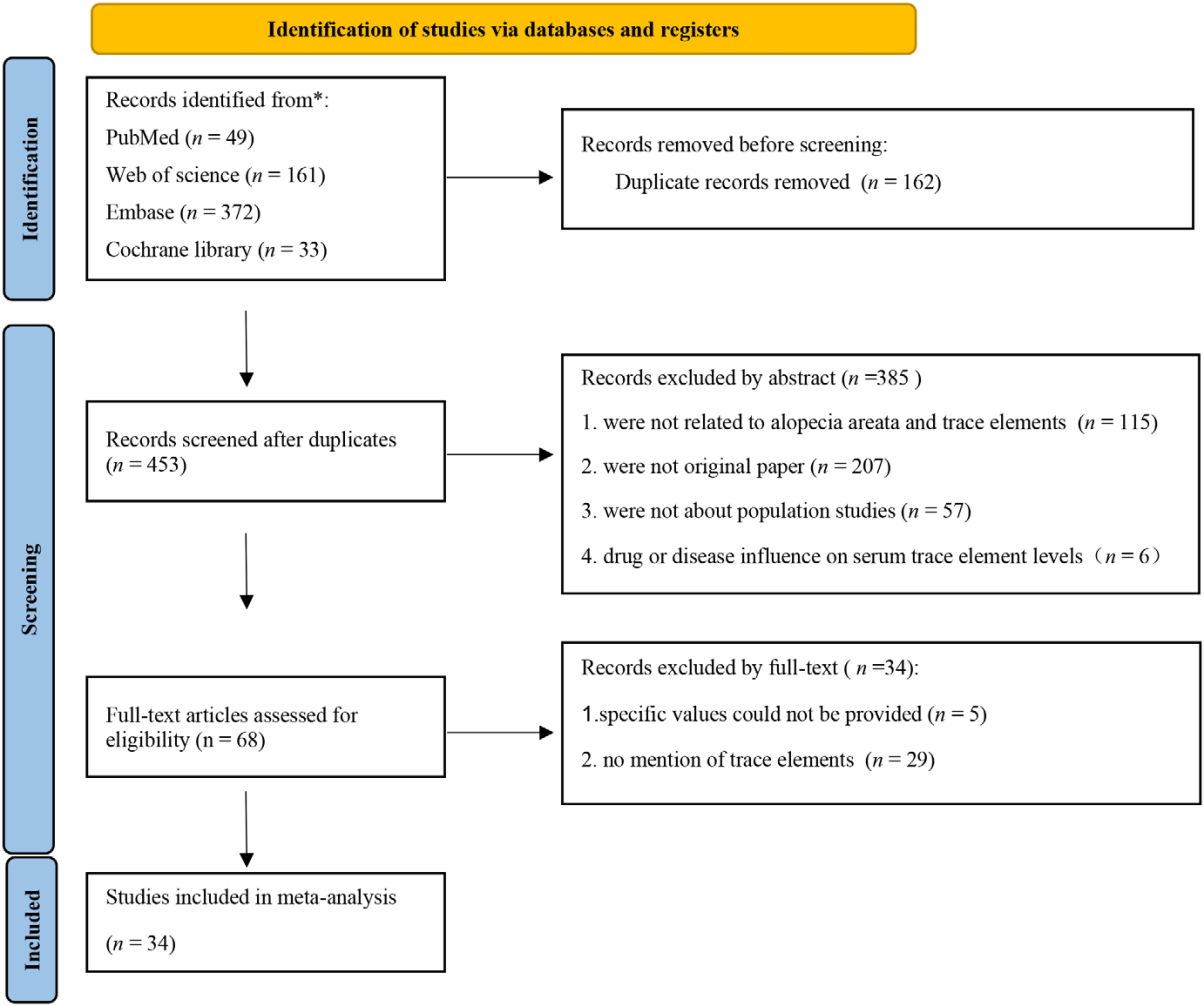
Flow chart of participant selection for this meta‐analysis.

We received 615 hits in total using the search method (Figure 1). After removing duplicates, 453 articles in total were deemed suitable for this meta‐analysis. 385 articles were excluded after reading the abstracts. Finally, 34 papers were included in the research after reading the entire text. There are six articles dealing with multiple trace elements [20, 21, 22, 23, 24, 25]. These studies included 4931 participants from 16 countries, which are Turkey (7 articles), India (5 articles), Egypt (4 articles), Iran (4 articles), America (2 articles), Arabia (2 articles), China (1 article), Israel (1 article), Japan (1 article), Korea (1 article), Nepal (1 article), Pakistan (1 article), Philippines (1 article), Serbia (1 article), Srinagar (1 article), and Thailand (1 article). We included a total of 10 cross‐sectional studies and 14 case–control studies (Table 1). According to the study quality assessment, 15 studies were judged to be of “high quality,” and another study was of low quality. Table 1 displayed the specifics of the included studies.

**TABLE 1:** Basic characteristics of studies included in the systematic review and meta‐analysis.

First author, year	Research setting	Sample size	Study type	Age (case group/health control group)	Male/Female	The type of trace elements	Study quality
Sabir Hasanbeyzade, 2024	Ankara, Turkey	82	Case–control study	26.85 ± 7/26.88 ± 6.95	82/0	Vitamin D	Low
Ahmed Ibrahim AbdElneam, 2024	Shaqra University	163	Case–control study	25 ± 3.9/23.8 ± 2.8	78/85	Vitamin D	High
Jovan Lalosevic, 2023	Serbia	64	Case–control study	5–57	37/27	Serum zinc	High
Saurabh Swaroop Gupta, 2023	Mullana, India	50	Case–control study	10–60	34/16	Vitamin D	Low
Adel Alsenaid, 2023	Dawadmi, Shaqra University, and the Department of Dermatology, Qassim University	119	Cross‐sectional	27.1 ± 9.1/27.4 ± 10.3	104/15	Vitamin D	High
Yamei Gao, 2022	Hefei, China	1252	Case–control study	31.28 ± 14.42/30.89 ± 13.0	738/514	Vitamin D	High
Kundak S., 2021	Turkey	212	Case–control study	0–18	110/102	Vitamin D	Low
Felix Paolo J. Lizarondo, 2021	Philippines	58	Cross‐sectional	19–65/19–64	20/38	Vitamin D	Low
Suchana Marahatta, 2019	Nepal	60	Case–control study	28.37 + 10.07	31/29	Vitamin D	Low
Mohamed I., 2019	Zagazig, Egypt	40	Cross‐sectional	—	—	Vitamin D	Low
Harsha Siddappa, 2019	India	200	Case–control study	5–60	130/70	Vitamin D	Low
Goknur Ozaydin‐Yavuz, 2019	Turkey	61	Case–control study	19–48/20–54	35/26	Serum copper and zinc	Low
Venkata Krishna Vamsi Gade, 2018	South India	90	Case–control study	32.73 ± 10.43/33.98 ± 8.48	28/62	Vitamin D	High
Sara Saniee, 2018	Iran	198	Case–control study	27.38 ± 11.94/29.54 ± 13.65	98/91	Vitamin D/serum zinc	Low
Mehmet Unal, 2018	Turkey	64	Case–control study	Male:12.4 ± 4.2/16.6 ± 0.8; Female:13.3 ± 4.4/ 16.5 ± 1.01	29/25	Vitamin D	High
Manju Daroach, 2018	India	60	Cross‐sectional	28.97 ± 9.96/31.17 ± 9.43	27/33	Vitamin D	High
Harsha Siddappa, 2018	America	60	Case–control study	11.13 ± 4.17/11.47 ± 4.42	34/26	Vitamin D	Low
Yasmeen Jabeen Bhat, 2017	India	85	Cross‐sectional	20.96 ± 1.91/21.37 ± 1.70	/	Vitamin D	Low
Seval Erpolat, 2017	Turkey	73	Cross‐sectional	20–50/20–51	44/29	Vitamin D	Low
Ruzica Z. Conic, 2017	Cleveland Clinic	756	Case–control study	35.54 ± 19.28/35.80 ± 15.56	230/526	Vitamin D	High
Rabia Ghafoor, 2017	Dermatology OPD, Jinnah Postgraduate Medical Centre, Karachi.	60	Case–control study	15–45	24/36	Vitamin D	Low
Ola Ahmed Bakry, 2016	Egypt	120	Case–control study	20–38/19–36	64/56	Vitamin D	Low
Nermeen S. A., 2016	Cairo, Egypt	100	Case–control study	7–44	78/22	Serum zinc	High
Kumpol Aiempanakit, 2016	Thailand	60	Cross‐sectional	Median age: 37/38	20/40	Serum zinc	Low
Nermeen S. A., 2015	Cairo, Egypt	60	Case–control study	19–50	36/24	Vitamin D	Low
Mahmud Mahamid, 2014	Israel	43	Case–control study	24.2 ± 12.3/27 ± 11.26	27/16	Vitamin D	High
Ladan Dastgheib, 2014	Iran	43	Case–control study	14–40	0/43	Serum copper and zinc	High
A. Aksu Cerman, 2014	Turkey	144	Cross‐sectional	32.21 ± 9.60/32.55 ± 9.78	90/54	Vitamin D	High
Soheila Nassiri, 2013	Loghman‐e‐Hakim and Shohada‐e‐Tajrish Clinic Dermatology	72	Case–control study	—	35/37	Vitamin D	Low
Min Seong Kil, 2013	Seoul, Korea	126	Cross‐sectional	36.61 ± 13.81/33.50 ± 10.50	—	Serum zinc	High
Mehdi Amirnia, 2013	Dermatology clinic of Tabriz Sina Hospital	54	Case–control study	66.27 ± 9.90/27.11 ± 5.55		Serum copper and zinc	Low
Yasmeen J. Bhat, 2009	Departments of Dermatology and Biochemistry, SKIMS Medical College Hospital, Srinagar	100	Cross‐sectional	6–60	68/32	Vitamin D/serum copper/zinc	High
R. Naginiene, 2004	Turkey	121	Case–control study	—	—	Vitamin D	Low
M. Tasaki, 1993	Japan	81	Case–control study	—	—	Serum copper and zinc	High

### 3.2 Vitamin D

**FIGURE 2:**
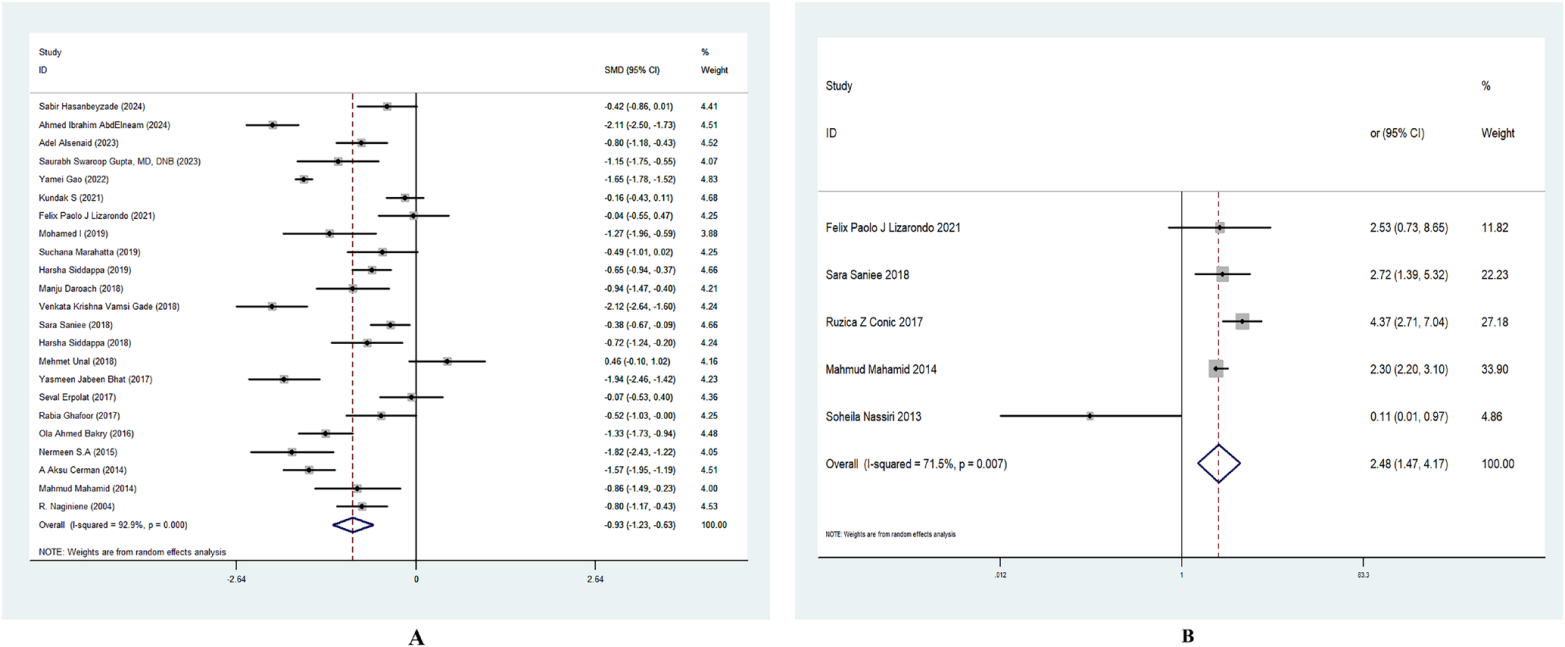
Meta‐analysis of the association between the risk of alopecia areata and (A) the level of vitamin D, (B) vitamin D deficiency. CI, confidence interval; OR, odd ratio; SMD, standard mean deviation.

For serum VD, 23 studies were selected, totaling 3404 participants. Only one study indicated the opposite of what the majority of studies demonstrated, with serum vitamin D levels in AA patients being considerably lower than in healthy controls. The heterogeneity test revealed a high level of heterogeneity (*I* ^2^ = 92.9%). Hence, a random‐effects model was utilized to aggregate the results. The results of meta‐analysis demonstrated a pooled SMD of −0.93 ng/mL, 95% CI of 0.168–0.747 (Figure 2A).

For the association between vitamin D deficiency and AA, 5 studies were selected, totaling 1127 participants. The heterogeneity test revealed a high level of heterogeneity (*I* ^2^ = 71.5%). The risk of AA was significantly increased by vitamin D insufficiency, according to the total pooled effect (OR = 2.48, 95% CI: 1.47–4.17) (Figure 2B).

### 3.3 Serum Zinc

**FIGURE 3:**
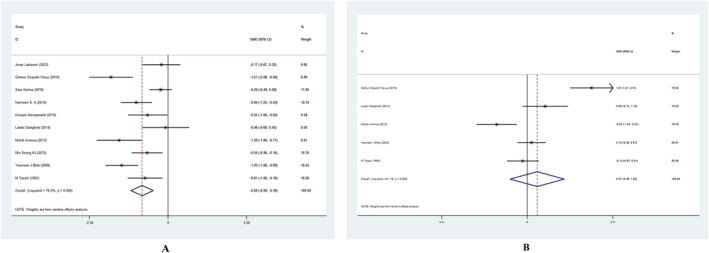
Forest plot of studies in serum (A) zinc, (B) copper for patients with alopecia areata versus healthy controls. CI, confidence interval; SMD, standard mean deviation.

For serum zinc, 10 studies were selected, totaling 878 participants. Based on every study, AA patients' serum zinc levels were considerably lower than those of healthy controls. The heterogeneity test revealed a high level of heterogeneity (*I* ^2^ = 76.5%). Hence, a random‐effects model was utilized to aggregate the results. The results of meta‐analysis demonstrated a pooled SMD of −0.69 μg/dL, 95% CI of −0.99 to −0.39 (Figure 3A).

### 3.4 Serum Copper

For serum copper, five studies were selected, totaling 339 participants. Two studies showed that serum copper levels were significantly lower in patients with AA than in healthy controls, and three studies illustrated the opposite results. The heterogeneity test revealed a high level of heterogeneity (*I* ^2^ = 91.1%). Hence, a random‐effects model was utilized to aggregate the results. The results of meta‐analysis demonstrated that there is no significant difference between the AA patients and controls in the levels of serum copper (SMD = 0.27 μg/dL, 95% CI of −0.49 to 1.04) (Figure 3B).

### 3.5 Subgroup Analysis

**FIGURE 4:**
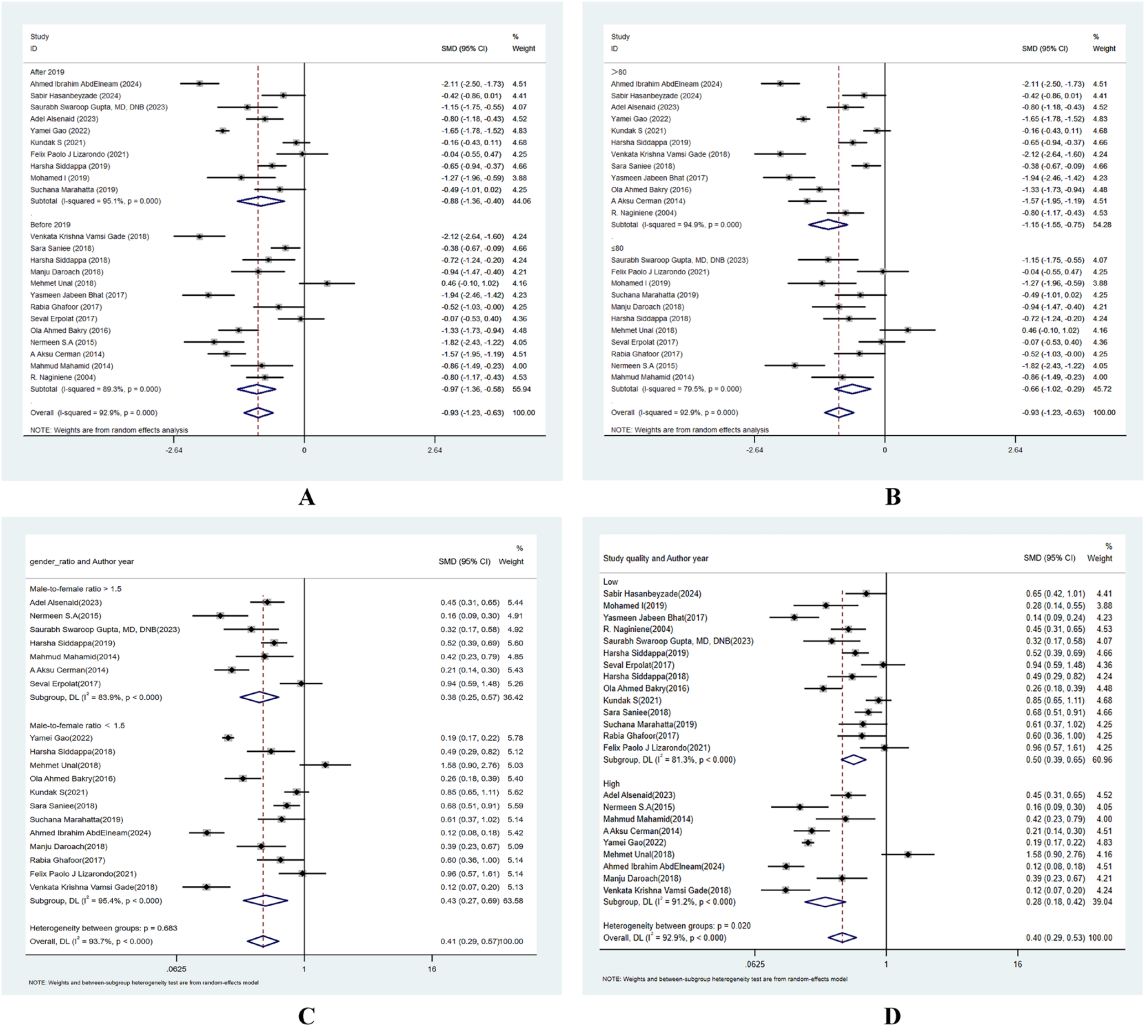
Subgroup analyses of the relationship between the risk of alopecia areata and the level of vitamin D. (A) publication year, (B) sample size, (C) gender ratio, and (D) study quality.

**FIGURE 5:**
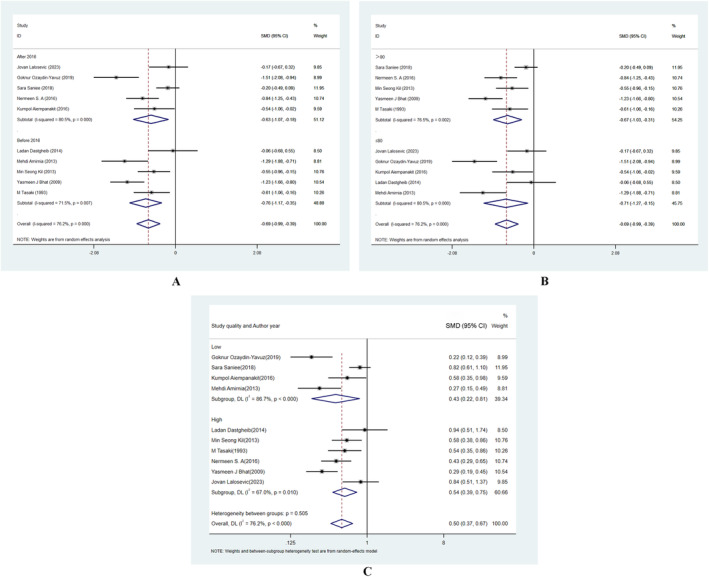
Subgroup analyses of the relationship between the risk of alopecia areata and the level of zinc. (A) publication year, (B) sample size, and (C) study quality.

In order to investigate potential reasons for the heterogeneity, we carried out extra subgroup analysis. For the serum VD, the subgroup analysis results demonstrated that heterogeneity was not caused by the publication year, sample size, male‐to‐female ratio and study quality (Figure 4A–4D). For the serum zinc, the subgroup analysis results demonstrated that heterogeneity was not caused by the publication year, sample size, and study quality (Figure 5A–5C).

### 3.6 Sensitivity Analysis and Publication Bias

**FIGURE 6:**
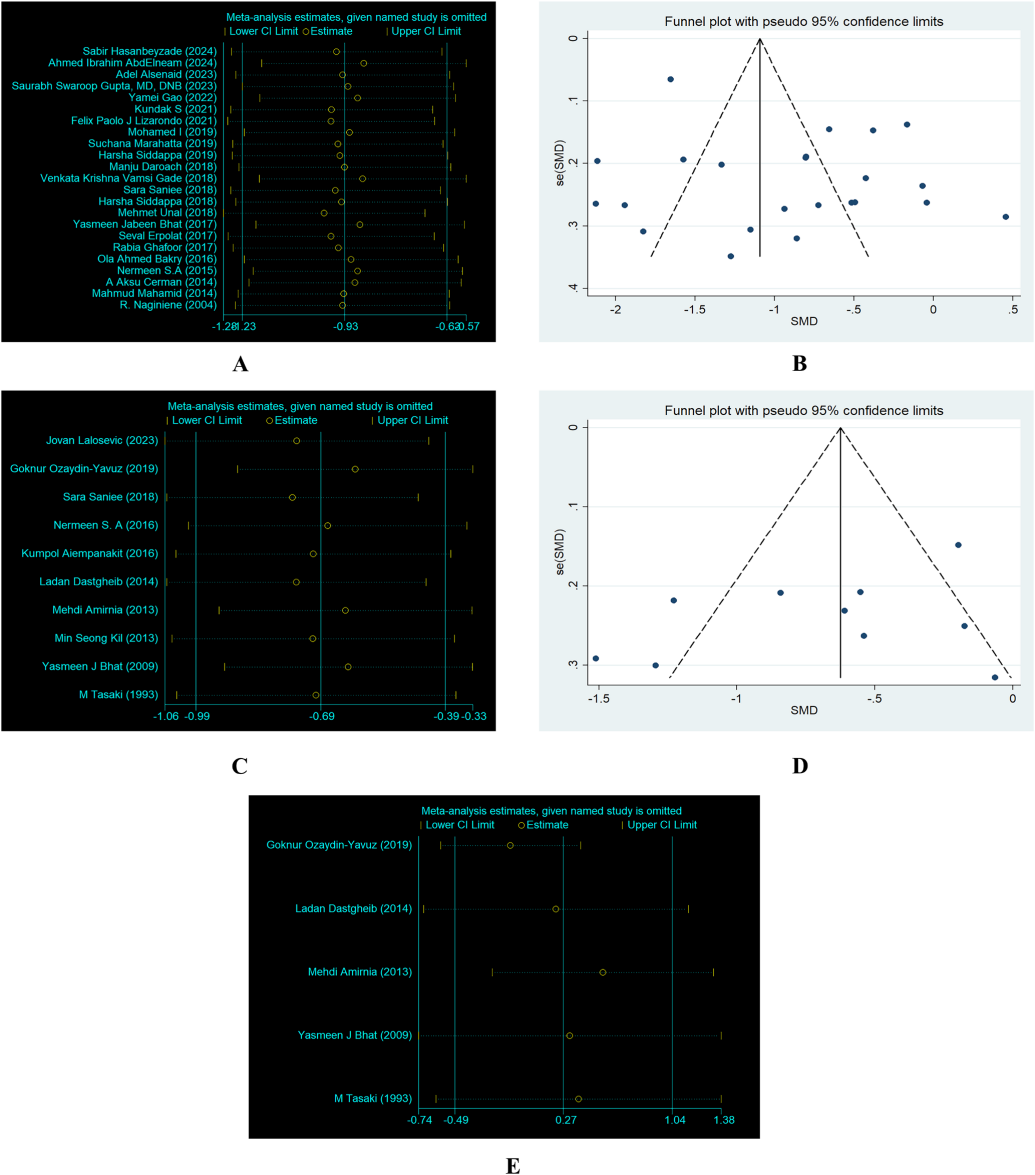
Sensitivity analysis and funnel plot of included studies. Vitamin D: sensitivity analysis (A) and funnel plot (B); zinc: sensitivity analysis (C) and funnel plot (D); copper: sensitivity analysis (E).

For the level of serum vitamin D, the overall results of the sensitivity analysis remained strong, demonstrating the durability of the conclusions. The primary findings remained substantial and strong even after removing any individual study (Figure 6A). The funnel plots demonstrated that research on serum VD and AA did not exhibit publication bias (Figure 6B). For the serum zinc, we found the similar results (Figure 6C,D). For the serum cupper, in sensitivity analysis, after most of the literature was deleted, the combined outcomes of the other studies were not statistically significant, suggesting that the original meta‐analysis's results were easily significantly changed due to the number of studies, and lacked robustness (Figure 6E).

## 4 Discussion

### 4.1 Key Findings

In the above systematic review and meta‐analysis, we extensively evaluated the existing data on serum trace elements (VD, zinc, and copper) levels and the risk of AA from 34 studies across 16 countries, involving a total of 4931 participants. The findings of the meta‐analysis suggested that serum levels of trace elements (VD and zinc) were significantly lower in AA patients compared to healthy controls, with VD deficiency identified as a risk factor for AA. However, the high heterogeneity among the studies included in this meta‐analysis significantly weakens the credibility of the aggregated evidence. We found no significant difference in serum copper levels between AA patients and healthy controls. This implies that low zinc and vitamin D levels in the blood may be risk factors for AA.

### 4.2 Comparison With Prior Meta‐Analyses and Interpretations

Previous studies exploring the association between serum trace elements and AA have mainly focused on the levels of iron/ferritin, zinc, copper, magnesium, and vitamin [24, 26, 27]. These studies have found that there may be a close association between serum trace elements and the occurrence of AA. But based on what we do know thus far, there is only one meta‐analysis that summarized all the literature published before April 30, 2016, exploring the changes in various serum trace element levels in AA patients [13]. The study found that serum zinc and selenium levels were lower in AA patients compared to healthy controls. There were no significant differences in serum copper, iron, ferritin, and magnesium levels between the AA group and the control group. However, this study did not assess the relationship between vitamin D levels and AA, and the search date is relatively outdated, failing to cover the latest research findings, which limits the reliability and persuasiveness of the results.

In contrast, we carried out a current review of the literature to May 2024, incorporating more studies and participants into our analysis (Table 1). Furthermore, we executed a systematic analysis that covered the relationship between multiple trace elements, including vitamin D, zinc, copper, and AA. Therefore, we believe our findings provide more current and comprehensive insights, contributing to a better public understanding of this topic.

Although we included variables such as publication year and sample size in the subgroup analysis to explore potential sources of heterogeneity, unfortunately, we did not identify any variables that could explain the heterogeneity. This suggests that other unmeasured or unreported variables maybe contribute more significantly to the heterogeneity, such as the duration of AA, severity, and disease activity status. It is important to interpret the results of studies on changes in serum trace element levels in AA patients with caution. Although most studies have found positive results, these studies are cross‐sectional or case–control and cannot establish a causal relationship between trace element levels and AA.

### 4.3 Interpretation of Findings

Although the potential pathophysiological mechanisms between trace elements and the risk of AA are not yet fully elucidated, increasing evidence suggests that trace element deficiencies are a significant risk factor for AA. In this research, we found that serum vitamin D deficiency or low levels are risk factors for AA, and the following are some potential explanations. As a secosteroid hormone, vitamin D is mostly produced by epidermal keratinocytes or obtained through diet. It plays several crucial roles in the human body, including helping maintain bone health, supporting immune system function, and regulating cell growth and differentiation [28]. 1,25(OH)2D and/or its receptor control a variety of skin processes, including the stimulation of differentiation, which includes the creation of the permeability barrier, the suppression of proliferation, the promotion of innate immunity, and the stimulation of the hair follicle cycle [29]. Vitamin D is essential for maintaining a normal hair cycle. Siddappa, Kumar, and Vivekananda [30] illustrated a significant negative association between vitamin D levels and the duration/severity of the disease. Furthermore, intralesional injection of vitamin D3 has been found to effectively improve AA [31].

Additionally, we found that low serum zinc levels may also be a potential risk factor for AA. Zinc is an vital trace element for the human health, promoting the recovery of hair follicles by preventing their degradation through the inhibition of nuclease activity [32]. Zinc is a crucial cofactor for DNA polymerase, involved in regulating cell cycle processes, and is closely related to DNA stability and repair. It ensures the normal division and function of hair follicle cells, contributing to normal hair growth and recovery [33]. Moreover, zinc has immunomodulatory effects that protect tissues with high cellular turnover from oxidative stress. It plays a critical function in keeping the hair cycle regular in people with hair issues [11]. Starace et al. found that oral zinc supplements improved hair loss and increased hair count [34].

It is well known that copper is a cofactor for tyrosinase, an enzyme that plays a crucial role in melanin synthesis [35]. Additionally, collagen and elastin, which are necessary for the structure and function of the skin and hair follicles, are synthesized in part by copper [36]. However, in this meta‐analysis, we found no significant difference in serum copper levels between the AA patients and healthy controls. This could be related to the small sample size, the mild severity of the disease in the included patients, and the short duration of the disease. Future large‐sample, multicenter longitudinal cohort studies are needed, with subgroup analyses based on the disease duration and severity, to fully explore the association between serum copper levels and the risk of AA.

### 4.4 Strengths and Limitations

There are various advantages to this meta‐analysis. Initially, we conducted a comprehensive and systematic evaluation of the current epidemiological evidence regarding the relationship between AA and serum levels of trace elements (VD, zinc, and copper). Second, we integrated data from 16 countries involving 4931 participants, which provides a wide coverage and strong population representativeness, enhancing the reliability and external validity of our conclusions. Additionally, we analyzed multiple trace elements (VD, zinc, and copper), offering a comprehensive perspective on the relationships between different trace elements and AA, thus enriching the content and significance of the research. Furthermore, the study found that serum VD and zinc levels were significantly lower in AA patients than healthy controls, providing important reference information for clinical practice and public health policy.

However, there are certain limitations. First, the meta‐analysis cannot address the potential confounding factors inherent in the original studies, despite most included studies having adjusted for major potential confounders. Second, the studies we included were all cross‐sectional or case–control studies. Longitudinal studies are needed to understand the long‐term effects of trace element levels on AA, better understand disease outcomes, and provide some reference for the causal relationship between them. Third, it is noteworthy that the meta‐analysis exhibited high heterogeneity among the studies, which may reduce the credibility of the evidence. Although we conducted subgroup analyses to identify potential sources of heterogeneity, due to limited data, we were unable to capture every important element that could be a role in the observed heterogeneity, such as age. Unfortunately, we could not determine the specific sources of heterogeneity. Fourth, due to the limitations of the original data, we were unable to further assess the association between serum trace element levels and disease severity or duration. We recommend that future studies systematically report these parameters to enhance the significance of the results. Finally, the findings related to copper levels remain inconclusive. This inconsistency may be attributed to variations in study populations, sample sizes, or measurement methodologies across the included studies. Further research is needed to clarify the role of this trace element in AA and its underlying mechanisms.

## 5 Conclusions and Future Outlook

In conclusion, our analysis provides the latest and most comprehensive evidence on the relationship between serum trace element levels and AA. We found that low serum VD and zinc levels may be risk factors for AA, which offers some reference for guiding clinical treatment. This suggests that measuring serum VD and zinc levels in AA patients can serve as indicators for assessing disease risk and severity, aiding in early identification and intervention. Supplementation with VD and zinc may become a potential treatment for AA. In the public health domain, promoting sufficient intake of VD and zinc through awareness and education could reduce the risk of AA.

Longitudinal research might be planned in the future to investigate the possible biological mechanisms and the causal association between the levels of zinc and VD and the development of AA. Researching the efficacy of different doses and forms of VD and zinc supplements in AA patients to determine the optimal supplementation regimen is also important. Developing and promoting dietary guidelines rich in VD and zinc can enhance public awareness of the importance of trace elements, particularly in areas or populations with a high incidence of AA.

## Author Contributions

Drafting the manuscript: R.W., Y.L., H.P.; critical revision of the manuscript for important intellectual content: Z.Y., Y.W., S.L.; responsibility for content: D.Y. All authors have read and agreed to the published version of the manuscript.

## Conflicts of Interest

The authors declare no conflicts of interest.

## Supporting information


Table S1.

## Data Availability

Data sharing is not applicable to this article as no new data were created or analyzed in this study.
