# Supplementary material for: Association Between Serum Trace Elements Level and Alopecia Areata: A Systematic Review and Meta‐Analysis
Source: J Cosmet Dermatol. 2024 Dec 30;24(1):e16740. doi: 10.1111/jocd.16740 (PMC11683520; doi:10.1111/jocd.16740)
Supplement: Supplementary file 1 — Table S1. [file JOCD-24-e16740-s001.docx]

**Table S1.** Search strategy: terms, databases and number of articles for review.

| **Databases** | **Strategy** | **Number of hits** | **Number imported into Endnote** |
| --- | --- | --- | --- |
| PubMed  Date (publication): Inception - 2024/5/12 | #1 Search ((((alopecia areata[MeSH Terms]) OR alopecia totalis[MeSH Terms]) OR alopecia universalis∗[Title/Abstract]) OR alopecia areata[Title/Abstract])  #2 Search (serum zinc [MeSH Terms]) OR (((((blood zinc [Title/Abstract]) OR serum Zn[Title/Abstract]) OR blood Zn [Title/Abstract]) OR zinc level [Title/Abstract]) OR Zn level [Title/Abstract])  #3 Search ("copper"[MeSH Terms] OR "serum copper"[Title/Abstract] OR "copper level"[Title/Abstract] OR "plasma copper"[Title/Abstract] OR "copper"[Title/Abstract])  #4 Search (vitamin D [MeSH Terms]) OR ((((((((cholecalcifero [Title/Abstract]) OR ergocalciferol [Title/Abstract]) OR calciferol [Title/Abstract]) OR calcidol [Title/Abstract]) OR 25-hydroxy vitamin D [Title/Abstract]) OR 25-hydroxyvitamin D [Title/Abstract]) OR 25-hydroxycholecalciferol [Title/Abstract]) OR 25-hydroxy cholecalciferol [Title/Abstract])  #5 #2 OR #3 OR #4  #6 #1 AND #5 | 6,389  2,569  157,672  21,230  180,696 | 49 |
| Web of Science  Date (publication): Inception - 2024/5/12 | #1 TS=(alopecia areata OR alopecia totalis OR alopecia universalis)  #2 TS=(serum zinc OR blood zinc OR serum Zn OR blood Zn OR zinc level OR Zn level)  #3 TS=(copper OR serum copper OR copper level OR plasma copper)  #4 TS=(vitamin D OR cholecalcifero OR ergocalciferol OR calciferol OR calcidol OR 25-hydroxy vitamin D OR 25-hydroxyvitamin D OR 25-hydroxycholecalciferol OR 25-hydroxy cholecalciferol)  #5 #2 OR #3 OR #4  #6 #1 AND #5 | 6,522  117,741  597,046  130,775  816,810 | 161 |
| EMBASE  Date (publication): Inception - 2024/5/12 | #1 ‘alopecia areata’:ab,ti OR ‘alopecia totalis’:ab,ti OR ‘alopecia universalis’:ab,ti  #2 'zinc'/exp OR zinc OR 'zn'  #3 'copper':ab,ti OR 'serum copper':ab,ti OR 'copper level':ab,ti OR 'plasma copper':ab,ti  #4 'vitamin d'/exp OR 'vitamin d$' OR cholecalciferol$ OR ergocalciferol$ OR calciferol OR calcidol OR 25$OH$D$ OR '25-hydroxy vitamin D$' OR '25-hydroxyvitamin D$' OR calcifediol OR '25-hydroxycholecalciferol' OR '25-hydroxy cholecalciferol'  #5  #2 OR #3 OR #4  #6 #1 AND #5 | 7,840  339,249  143,820  216,736  655,620 | 372 |
| Cochrane library  Date (publication): Inception - 2024/5/12 | #1 alopecia areata OR alopecia totalis OR alopecia universalis  #2 “zinc”/exp OR zinc OR “zn”  #3 copper OR serum copper OR copper level OR plasma copper  #4 “vitamin d”/exp OR “vitamin d$” OR cholecalciferol$ OR ergocalciferol$ OR calciferol OR calcidol OR 25$OH$D$ OR “25$hydroxy vitamin D$” OR “25$hydroxyvitamin D$” OR calcifediol OR “25$hydroxycholecalciferol” OR “25$hydroxy cholecalciferol”  #5 #2 OR #3 OR #4  #6 #1 AND #5 | 838  8,369  2,788  21,553  30,815 | 33 |
| **TOTAL RESULTS** |  |  | 615 |
